# Exploring the effects of head movements and accompanying gaze fixation switch on steady-state visual evoked potential

**DOI:** 10.3389/fnhum.2022.943070

**Published:** 2022-09-12

**Authors:** Junyi Duan, Songwei Li, Li Ling, Ning Zhang, Jianjun Meng

**Affiliations:** ^1^Department of Mechanical Engineering, Shanghai Jiao Tong University, Shanghai, China; ^2^Shanghai Electro-Mechanical Engineering Institute, Shanghai, China; ^3^National Research Center for Rehabilitation Technical Aids, Beijing, China

**Keywords:** steady-state visual evoked potential, brain-computer interface, head movements, virtual reality, gaze fixation switch

## Abstract

In a realistic steady-state visual evoked potential (SSVEP) based brain-computer interface (BCI) application like driving a car or controlling a quadrotor, observing the surrounding environment while simultaneously gazing at the stimulus is necessary. This kind of application inevitably could cause head movements and variation of the accompanying gaze fixation point, which might affect the SSVEP and BCI’s performance. However, few papers studied the effects of head movements and gaze fixation switch on SSVEP response, and the corresponding BCI performance. This study aimed to explore these effects by designing a new ball tracking paradigm in a virtual reality (VR) environment with two different moving tasks, i.e., the following and free moving tasks, and three moving patterns, pitch, yaw, and static. Sixteen subjects were recruited to conduct a BCI VR experiment. The offline data analysis showed that head moving patterns [*F*(2, 30) = 9.369, *p* = 0.001, effect size = 0.384] resulted in significantly different BCI decoding performance but the moving tasks had no effect on the results [*F*(1, 15) = 3.484, *p* = 0.082, effect size = 0.188]. Besides, the canonical correlation analysis (CCA) and filter bank canonical correlation analysis (FBCCA) accuracy were better than the PSDA and MEC methods in all of the conditions. These results implied that head movement could significantly affect the SSVEP performance but it was possible to switch gaze fixation to interact with the surroundings in a realistic BCI application.

## Introduction

Brain-computer interface (BCI) is a novel technology that directly bridges the brain and external devices without relying on the pathway between the spinal cord and muscles (Wolpaw, 2013; He et al., 2020). BCI could assist in reconstructing motor function for people with severe motor disabilities like stroke, neuromuscular dystrophy, and amyotrophic lateral sclerosis (Cincotti et al., 2008; Ang and Guan, 2015; Borgheai et al., 2020). Steady-State Visual Evoked Potential (SSVEP) is the periodic electrophysiological response to repetitive visual stimulus. It can be detected over the occipital region (Müller-Putz et al., 2005; Zhu et al., 2010). SSVEP-based BCIs have been widely studied in recent years because of SSVEP’s high signal-to-noise ratio (SNR) and excellent user adaptability. Thus, successful applications such as BCI spellers (Chen et al., 2015b; Xu et al., 2016; Nakanishi et al., 2017) have been intensively demonstrated during the past decades.

Besides applications in BCI spellers, the SSVEP-based BCIs also gained attention in physical/virtual object interactions such as the wheelchair and robotic arm control due to their high decoding accuracy (Li et al., 2013; Chen et al., 2020). Apart from displaying visual stimulation by a computer screen, virtual reality (VR), a new display technology, has been increasingly studied in the SSVEP-based BCI for better immersion and interaction with the surrounding environment (Lécuyer et al., 2008; Coogan and He, 2018). Koo et al. (2015) validated the improved performance in an SSVEP-controlled VR maze game compared to the case in a monitor display. Armengol-Urpi and Sarma (2018) investigated the application of movie playback control in VR with visual stimuli. Wang et al. (2018) designed a wearable SSVEP-BCI, which navigated quadcopter flight in a 3D virtual environment. Utilizing VR in SSVEP-based BCI has shown its superiority compared to conventional displays and has become increasingly popular. However, the effect of changing the stimulus presentation environment on SSVEP signals deserves further investigation.

Unlike traditional BCIs that present tasks on flat screens, head movement frequently occurs in interactive BCI VR applications. Head movement is apparent, especially during real-time control tasks like driving cars and interacting with people in the game when subjects need to observe the surrounding environment and issue commands simultaneously. Several studies have explored the effects of movement on SSVEP-based BCI with visual stimuli displayed on the screen. Lin et al. (2014) assessed the quality of SSVEPs for people walking on a treadmill, but the movement was a coupling of the sub-motions of various parts. Kanoga et al. (2019) analyzed the effects of head movement on signal characteristics and classification performance by gazing at a single moving stimulus on an ultra-widescreen. However, the sight was limited to the center of the stimulus, which could not be applied in real-time control.

Moreover, subjects might fix their gaze on one external object rather than the stimulus in the same visual field to keep abreast of the surroundings. Some studies called this phenomenon visual selective attention (Müller and Hillyard, 2000; Zhang et al., 2010). They have shown that the SSVEP amplitude of unattended flicker stimulus was smaller than the attended one’s; Zhang et al. (2019) found a downward trend in the SSVEP response with increasing gaze distance from the center. Thus, switching gaze between the stimulus and the surrounding environment might affect the SSVEP-based BCI performance. However, the experiment was limited to the steady gaze, while in a practical BCI, gaze fixation could frequently switch from the stimulus to the environment and vice versa. VR technology provided a powerful and unique tool to investigate the effect of head movement and accompanied gaze fixation switch separately. As a result, it is worth designing a practical SSVEP-BCI in VR to discuss the effects of head movement and accompanied gaze fixation switch. To the best of our knowledge, no study has discussed these before.

By designing a new ball tracking paradigm, this study found that the head movement could significantly affect the BCI performance. However, no differences were found between the yaw and pitch movement. Moreover, the gaze fixation switch didn’t show a significant influence on BCI performance, which indicated that it was possible to interact with the surrounding environment while doing the SSVEP task. This article is organized as follows: Section “Materials and methods” is the materials and methods containing experimental setup, paradigm, and data processing; Section “Results” covers the results of pose data, signal characteristics, and target identifications; Section “Discussion” is the discussion, and Section “Conclusion” is the conclusion of this article.

## Materials and methods

### Experimental setup

**FIGURE 1:**
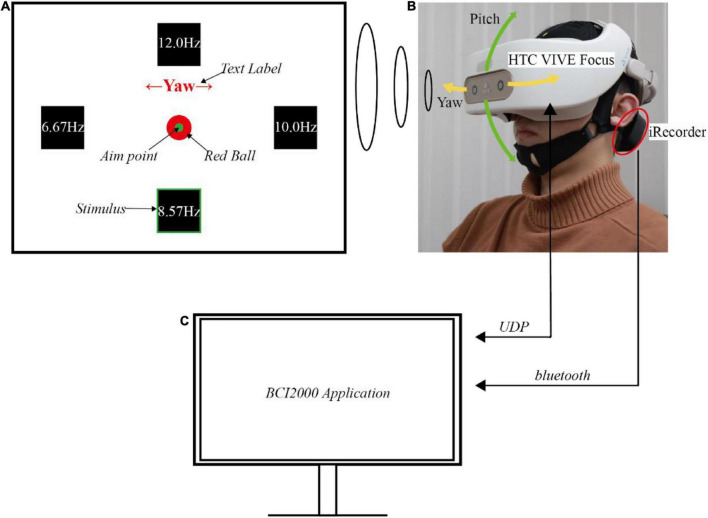
The experimental setup. **(A)** Illustration of the user interfaces in virtual reality. **(B)** Demonstration of a subject performing the experiment. **(C)** Illustration of the computer and its wireless communication.

The SSVEP-based BCI system’s hardware included an EEG acquisition device, a head-mounted device (HMD), and a computer. The experiment was executed in an electromagnetic and acoustic shielding room to reduce ambient noise and distraction. Figures 1A,B show the scenario of a participant performing this experiment.

The EEG data were recorded with iRecorder W16 (Shanghai Idea-Interaction Tech., Co., Ltd.), a wireless and portable commercial EEG acquisition device. This portable acquisition device guaranteed the EEG signal quality during subjects’ movement since the potential noise caused by the wire movement was avoided. Six signal electrodes (PO3, POz, PO4, O1, Oz, and O2) over the occipital area were used to decode the SSVEP, while FCz and CPz were used as the ground and reference electrodes, respectively, according to the international 10−20 system. The analog signals were sampled at 500 Hz with a compact wireless amplifier, magnetically attached to the back of the cap (the black gadget shown in Figure 1B). The amplifier transmitted the raw EEG signals through the Bluetooth serial module plugged onto the computer’s USB port, from which the computer can read the data for further signal processing.

The HMD used in this experiment was HTC VIVE Focus (HTC Corporation), an all-in-one VR machine with no wire linked to the computer. This wireless device also helped the subject move freely without wires pulling. This HMD could display the virtual scene with 2880 × 1600 binocular resolution, a maximum of 75 Hz refresh rate, and a 110° field of view (FOV). For operational stability, the refresh rate in this experiment was locked to 60 Hz. An additional belt was tied to the HMD along the central axis from front to back. This belt helped reduce the pressure of the HMD on the cheek and secure the HMD on the head, eliminating the relative movement of HMD to the head. The virtual scene was constructed using Unity 3D software, a game engine usually used in VR. The scene consisted of four parts: four stimuli, a gaze fixation point, a text label, and a ball (Figure 1A). The stimuli’s flickering frequencies were selected as 6.57 Hz, 8.57 Hz, 10 Hz, and 12 Hz, representing flicker once every 9, 7, 6, and 5 frames. They were four squares on the left, down, right, and upsides of the FOV.

For each horizontal stimulus, the off-center angle was 16.7 degrees. While for each vertical stimulus, the angle was 14 degrees, which was smaller than the horizontal one to match the human’s natural view angle with broader horizontal FOV. The angles were selected to balance natural gaze of the subject and the greatest separation of the stimuli. The gaze fixation point was a green dot in the center of the four stimuli used to track the ball’s movement. The stimuli and the fixation point were attached and bound together with the virtual camera, which was denoted as the virtual eye. This virtual camera kept the relative position of the stimuli and fixation point in the FOV unchanged. Moreover, because humans tended to look downward at around 15° in a natural gazing state, all the components were located below the center of the FOV, which helped the user gaze at the fixation point naturally (Mon-Williams et al., 1998).

There were three moving patterns: static, yaw, and pitch. A text label was shown on the upper side, indicating the current moving pattern, and was only visible before each trial. The text was one of Static, ←Yaw→, and ↑Pitch↓, denoting each moving pattern, with red color and sufficiently prominent font size. The red ball was displayed as a target, and it instructed the user to follow it and generate a correct head movement. The head pose data was collected using WaveXR (HTC Corporation) software development kit to determine whether the subjects correctly followed the instructions of moving their heads. The HMD’s quaternions in each timestamp were converted to Euler angles in the customized Unity application and sent to the computer via UDP. Figure 1B also showed the representation of Euler angles.

An open-source BCI software platform, BCI2000, was running on the computer (Figure 1C; Schalk et al., 2004). It recorded the EEG and pose data, synchronized them, and saved them on the computer. Furthermore, by keeping the computer and the HMD in the same local area network, the customized BCI2000 application also controlled the contents displayed in the HMD in different phases by UDP.

### Experimental paradigm

Sixteen subjects (10 males and 6 females; age: 24.0 ± 4.6 years) with normal or corrected to normal vision participated in this experiment. Thirteen of them were naïve to the SSVEP-based BCI experiments. They were all fully informed of the procedure and signed the informed consent before the experiment. The Institutional Review Board of Shanghai Jiao Tong University approved all procedures and protocols.

Subjects needed to gaze at one of the four stimuli and move their head simultaneously to figure out the influence of head movement and its accompanying distraction and be close to the practical application. There were two types of moving tasks: one was the following task, and the other was a free one. In the following task, the red ball would move around the virtual camera vertically or horizontally at a constant speed of 10°/s or maintain still according to the moving pattern. Subjects needed to move their head to covertly pay attention to the ball’s movement and follow it until it stopped. Therefore, subjects should focus on the flickering stimuli and spare limited attention to the moving ball to follow it in this task.

Consequently, it was necessary to design another task to explore the influence of the gaze fixation switch due to the following movement. Correspondingly in the free task, the ball was still, and subjects needed to rotate their heads voluntarily according to the indicated pattern and tried to keep the same speed as the following task. If subjects moved out of boundaries, the green frame of the gazed stimulus would turn red to inform the subject. A practice session before the whole experiment was conducted until the subjects could be fully acquainted with the moving details. This procedure usually lasted for at most a couple of minutes.

**FIGURE 2:**
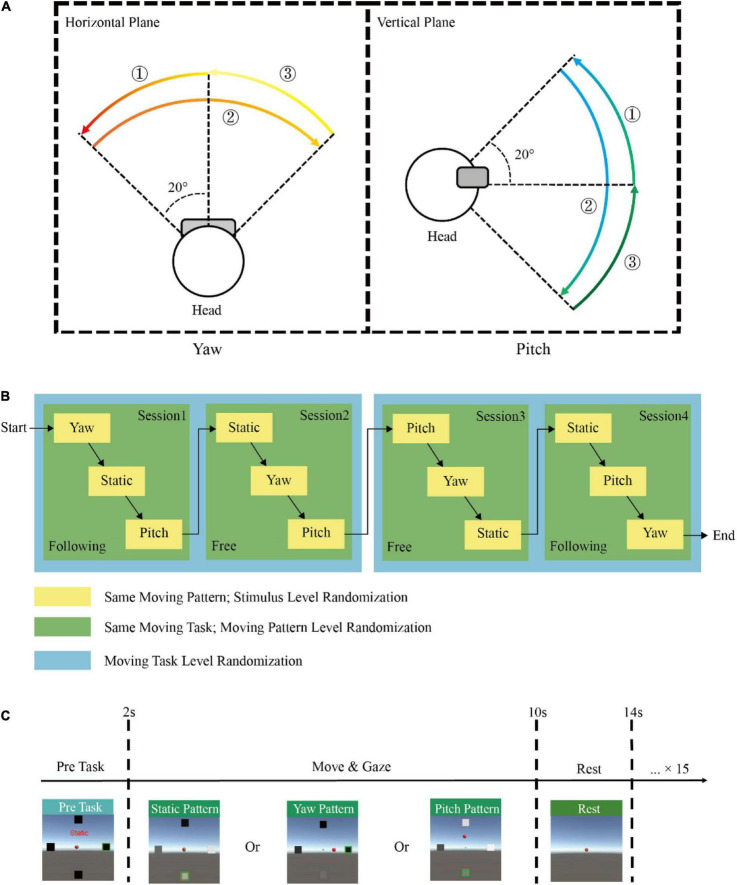
Experimental paradigm. **(A)** The moving range and orders (encircled numbers) when a subject performs yaw and pitch moving patterns. **(B)** An example of the experimental protocol by the block randomization design. **(C)** The demonstration of one single trial in this experiment.

There were three moving patterns in each task: static, yaw, and pitch. The static pattern was set as a baseline with only the SSVEP task but no head movement. For the yaw pattern, subjects needed to rotate their heads horizontally in a free or following way according to the indicated cue. First, subjects should rotate their heads from the origin (0°) to −20° on the left side, turn to +20° on the right side and return to the origin in each yaw trial. Similarly, for the pitch pattern, subjects should nod their heads vertically from the origin to −20° on the upside, then turn to +20° on the downside and return to the origin. The moving range and orders (encircled numbers) were shown in Figure 2A.

The experiment for each participant was divided into four sessions with two following task sessions and two free task sessions. Each session consisted of three runs, and one moving pattern was randomly assigned in each run. Subjects would take off the HMD and rest for 5 mins between each session. Each run included sixteen trials with the same moving task and pattern but different stimuli targets. A 2 mins break was set between each run. The moving task, moving pattern, and gazed stimulus were all in pseudo-random order to accomplish full block randomization. Figure 2B showed an example of the experimental protocol by block randomization. Therefore, a participant would repeat eight times for each condition, and the total number of trials obtained from each participant was 196 (2 moving tasks × 3 moving patterns × 4 frequencies × 8 repeated times). At the beginning of each trial, the four stimuli would appear, highlighting the target stimulus with a green box. Subjects needed to fix their gaze at the indicated stimulus and minimize gaze shifting during the task. The text label would be visible for 2 s to remind subjects of the current moving pattern. Then the stimuli started to flicker, and subjects needed to maintain their gaze and perform the corresponding moving task simultaneously for 8 s. Subjects were asked to avoid blinking during the stimulation to avoid ocular artifacts. In addition, body movement, swallowing, and gnash were not allowed to capture only head movement. After the 8th s, the stimuli disappeared, and subjects could rest for 4 s until the next trial started. The paradigm of one trial was shown in Figure 2C.

### Data processing

#### Preprocessing

The EEG signal processing was implemented in Matlab using the EEGLAB toolbox and customized scripts (Delorme and Makeig, 2004). The EEG data was firstly bandpass filtered from 3 to 30 Hz using a Hamming windowed sinc FIR filter. According to previous successful studies (Nakanishi et al., 2017), the latency of the BCI system and human vision system was estimated at around 140ms. Hence, the EEG data epoch was segmented from 0.14 s to 8 s, where 0 s represented the stimulus onset. The HMD’s Euler angle data was also segmented from −1 s to 8 s, with the baseline removed by subtracting the mean of amplitudes from −1 s to 0 s. There was an additional screening procedure to remove the artifact and erroneous trials before further processing. If a trial met any of the following five criteria, it would be manually excluded. The criteria were: (1) the maximum absolute amplitude exceeded the mean plus five standard deviations; (2) the actual moving pattern was not correct, e.g., pitching in a yaw trial; (3) the moving speed was too fast or slow, namely completing a cycle of movement with a period deviating from the mean more than 1.5 s; (4) hurry or delay, was that moving before the task start or didn’t move after 1 s of the stimulus onset; (5) the moving direction was not independent with irrelevant angle change larger than 7 degrees. Fifty-nine trials were finally excluded, with 1.88% of the total trials.

#### Signal characteristics

The wide-band signal-to-noise ratio (SNR) was calculated to compare the signal quality of SSVEP responses in different conditions (Liu et al., 2020). The whole data (0.14−8 s) were used to represent the overall signal properties in one trial. First, the power spectra were calculated by using the square value of the fast Fourier transform (FFT) of EEG signals. The wide-band SNR was defined as the ratio of the added power spectra of multiple harmonics to the total power from zero to the Nyquist frequency, excluding the power of harmonics, as:


(1) $$\begin{array}{c} S\,N\,R=10\,l\,o\,g_{10}\,\left(\frac{\sum_{k=1}^{k=N_{h}}P\,\left(k\cdot f\right)}{\sum_{f=0}^{f=\frac{f_{s}}{2}}P\,\left(f\right)-\sum_{k=1}^{k=N_{h}}P\,\left(k\cdot f\right)}\right) \end{array}$$

where *N*_*h*_ denotes the number of harmonics, *P*(*f*) denotes the power spectrum at the frequency of *f*, *f*_*s*_ is the sampling rate and *f*_*s*_/2 represents the Nyquist frequency. Comparing the classification accuracies using different numbers of harmonics in the preliminary experiments showed no difference beyond the second harmonic. Therefore in this study, *N*_*h*_ was set to 2. The wide-band SNRs of six channels were averaged to obtain the outcome of a single value.

#### Feature extraction and classification

To evaluate the effect of head movement on SSVEP tasks’ performance, calibration-free algorithms, including power spectral density analysis (PSDA), minimum energy combination (MEC), canonical correlation analysis (CCA), and filter bank canonical correlation analysis (FBCCA) were adopted to extract useful features and subsequently obtain the classification accuracy. The design principle of the algorithms is to extract response features maximally from EEG signals **X**_*N*_*c*_×*N*_, where *N*_*c*_ was the number of channels, *N* was the number of sampling points. In MEC and CCA, reference signals ${\text{Y}}_{2\times N_{h}\times N}^{i}$ were used as a template, which was usually the combination of sin and cos waves of a stimulus frequency *f*_*i*_, where *N*_*h*_ was the number of harmonics:


(2) $$\begin{array}{c} {\text{Y}}_{2\times N_{h}\times N}^{i}=\left(\begin{array}{c} \text{sin}\,\left(2\,\pi \,f_{i}\,\text{t}\right)\\ \text{cos}\,\left(2\,\pi \,f_{i}\,\text{t}\right)\\ \begin{array}{c} \text{sin}\,\left(4\,\pi \,f_{i}\,\text{t}\right)\\ \text{cos}\,\left(4\,\pi \,f_{i}\,\text{t}\right)\\ \begin{array}{c} \ldots \\ \text{sin}\,\left(2\,\pi \,N_{h}\times f_{i}\,\text{t}\right)\\ \text{cos}\,\left(2\,\pi \,N_{h}\times f_{i}\,\text{t}\right) \end{array} \end{array} \end{array}\right) \end{array}$$

In the following, X was the EEG signal mentioned above, and its sub-indexes *N*_*c*_, *N* were omitted for simplicity. Y represented the reference signals of frequency *f*_*i*_, its sub-indexes 2×*N*_*h*_, *N* and i were omitted as well.

The target class τ could be identified based on the maximum feature value obtained in each algorithm:


(3) $$\begin{array}{c} \tau =\underset{i}{\text{argmax}}\rho_{i},i=1,2,\ldots ,N_{f} \end{array}$$

where, ρ_*i*_ is the feature in each frequency, *N*_*f*_ was the number of stimuli classes.

##### Power spectral density analysis

Because of the periodicity of SSVEP signals, the intuitive idea was to find the response frequency with the maximum energy. PSDA was realized by using the fast Fourier transform to calculate the power spectral density (PSD) of the EEG signals (Cheng et al., 2002). It has been widely used because of its simplicity and high efficiency. The frequency response corresponding to the maximum power spectral density was considered as the target frequency:


(4) $$\begin{array}{c} \rho_{i}=P\,S\,D\,\left(f_{i}\right),i=1,2,\ldots ,N_{f} \end{array}$$

##### Minimum energy combination

Minimum energy combination was first proposed by Friman et al. (2007) as an autonomous, multiple channel detection method. MEC firstly removed SSVEP potentials from the electrode signals to obtain the nuisance signals $\tilde{\text{X}}$:


(5) $$\begin{array}{c} \tilde{\text{X}}={\text{X}}^{T}-{\text{Y}}^{T}\,{\left({\text{YY}}^{T}\right)}^{-1}\,{\text{YX}}^{T} \end{array}$$

The next step was to find a spatial filter **W**, to minimize the energy of nuisance signals by solving the optimization problem:


(6) $$\begin{array}{c} \min_{W}{∥{\tilde{\text{X}}}^{T}\,\text{W}∥}^{2}=\min_{W}{\text{W}}^{T}\,\tilde{\text{X}}\,{\tilde{\text{X}}}^{T}\,\text{W} \end{array}$$

The problem was solved by finding the eigenvalues and eigenvectors of the symmetric matrix $\tilde{\text{X}}\,{\tilde{\text{X}}}^{T}$. By selecting the eigenvalues (λ_1_ < … < λ_*Ns*_) in ascending order, **W** consisted of the corresponding weight vectors (**v**_1_, …,**v**_*Ns*_):


(7) $$\begin{array}{c} \text{W}=\left[\frac{v_{1}}{\lambda_{1}}\,\,\cdots \,\,\frac{v_{N_{s}}}{\lambda_{N_{s}}}\right] \end{array}$$

where *N*_*s*_ was the number of selected channels, based on how much nuisance signals should be discarded. In this study, 90% of the nuisance signals energy was discarded. Hence, the signals that removed noise for each channel could be obtained:


(8) $$\begin{array}{c} \text{S}={\text{X}}^{T}\,\text{W} \end{array}$$

The feature value ρ_*i*_ could be calculated based on signal and noise power estimation for each reference signal:


(9) $$\begin{array}{c} \rho_{i}=\frac{1}{N_{s}\,N_{h}}\,\sum_{l=1}^{N_{s}}\sum_{k=1}^{N_{h}}\frac{\hat{P_{k,l}}\,\left(f_{i}\right)}{{\hat{\sigma_{k,l}}}^{2}\,\left(f_{i}\right)},i=1,2,\ldots ,N_{f} \end{array}$$

The SSVEP signal power $\hat{P_{k,l}}\,\left(f_{i}\right)$ could be estimated by:


(10) $$\begin{array}{c} \hat{P_{k,l}}\,\left(f_{i}\right)={∥{\text{Y}}_{k}\,{\text{S}}_{l}∥}^{2} \end{array}$$

where **Y**_*k*_ is the *k*th harmonic component of ***Y***, **S**_*l*_ is the signal of the *l*th channel.

The noise power ${\hat{\sigma_{k,l}}}^{2}\,\left(f_{i}\right)$ could be obtained by fitting signals **S**_*l*_ with auto-regressive models *AR*(*p)* using the Yule-Walker method:


(11) $$\begin{array}{c} {\hat{\sigma_{k,l}}}^{2}=\frac{\pi \,N}{4}\,\frac{{\hat{\sigma }}^{2}}{{\left|1+\sum_{j=1}^{p}\alpha_{j}\,\exp\left(-\frac{2\,\pi \,i\,j\,k\,f}{F\,s}\right)\right|}^{2}} \end{array}$$

Here, ${\hat{\sigma }}^{2}$ was the estimation of the white noise when deriving the *AR*(*p)* process, α_*j*_ was the model parameters, and the model order was set *p* = 15 in this study based on previous research (Davila et al., 1998; Friman et al., 2007).

##### Canonical correlation analysis

Canonical correlation analysis was a multivariate statistical method to maximize the correlation between two multidimensional variables (Lin et al., 2006; Bin et al., 2009). It had been widely used for its high accuracy, robustness, and ease of use. CCA tried to find the best spatial projection vector **w** and **v**, to maximize the correlation between the EEG signals **X** and the reference signals ***Y***:


(12) $$\begin{array}{c} \rho_{i}=\underset{w,v}{m\,a\,x}\frac{w^{T}\,\mathrm{XYv}}{\sqrt{w^{T}\,{\mathrm{XX}}^{T}\,{\mathrm{wv}}^{T}\,{\mathrm{YY}}^{T}\,v}},i=1,2,\ldots ,N_{f} \end{array}$$

The sliding window method was used with a step of 0.2 s to simulate the online detection process and window length varied from 0.8 s to 2.0 s for each method. Signals in each window gave out a result and the accuracy was calculated to divide the number of correct results by the total number of classification results.

##### Filter bank canonical correlation analysis

Filter bank canonical correlation analysis was an extension of CCA methods by utilizing harmonic frequency components to improve the detection of SSVEPS (Chen et al., 2015a). It has shown its superiority compared with CCA, especially under the condition of a high amount of stimuli. FBCCA used multiple filter banks to decompose the EEG signals into several sub-band components and applied CCA on each component. The final result was obtained by weighting each correlation coefficient and adding them together.

In this study, the number of harmonics *N*_*h*_ were selected as 2 based on standard CCA formulation. The M3 method in Chen et al. (2015a) was adopted by using aI-type Chebyshev filter, and the parameters were optimized by the first 25% trials in each condition. The number of filter banks N and the weight parameters a and b were determined by a grid search in the parameter space of [1:1:9], [0:0.25:2], and [0:0.25:1] respectively. Finally, *N* = 5, *a* = 1.25, *b* = 0.25 were selected.

### Statistical analysis

Three-way repeated measures analysis of variances (ANOVA) was applied to the wide-band SNR (2 moving tasks × 3 moving patterns × 4 frequencies) to test the effect of different factors. Besides, four-way repeated measures of ANOVA were applied to the spectrum amplitude (2 moving tasks × 3 moving patterns × 4 frequencies × 2 harmonics) and SSVEP decoding accuracy (2 moving tasks × 3 moving patterns × 4 frequencies × 4 algorithms) to explore the differences. All of the data were tested with sphericity and corrected using the Greenhouse-Geisser method if the sphericity was violated. In addition, the significances due to multiple comparisons were corrected using Bonferroni correction in *post hoc* tests.

## Results

### The Euler angles of head posture

**FIGURE 3:**
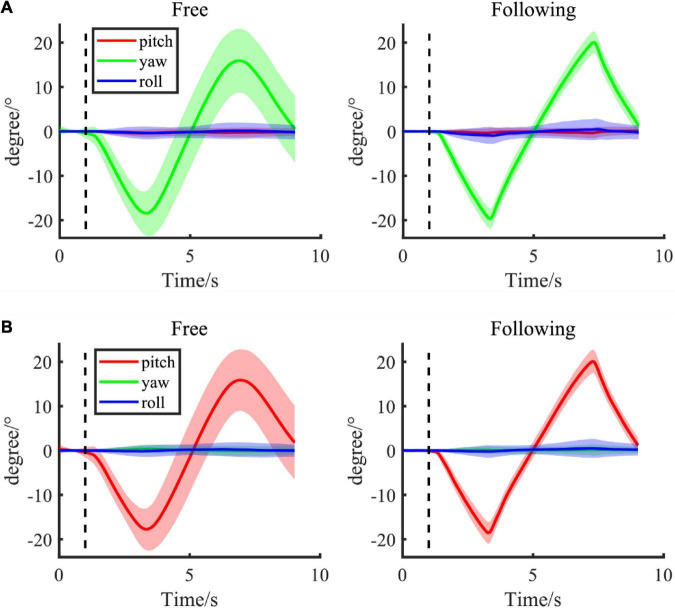
**(A,B)** Group average of Euler angle change in different following tasks (left column: free task; right column: following task) with different moving patterns, including yaw and pitch. The shadow area presented the standard error of the group population.

The mean Euler angles in different moving tasks and patterns throughout the experiment were shown in Figure 3. Figure 3A showed the Euler angles changed when subjects were yawing their heads in both free and following moving tasks, while Figure 3B displayed the pitching results. Most subjects followed the target very well at a uniform speed, and the speed was well controlled even in the free moving task without the indicating moving ball. Their head movements were smooth and similar to the angle changes in the following task. Moreover, subjects tended to move more continuously at the turning back point in the free moving task, without a sudden speed change. However, the moving range in the free moving task was smaller than the range in the following task for about 3−4 degrees on both sides. This difference might be due to the varying arrival times on both sides. Therefore, the amplitudes might be smoother when averaging them together. Moreover, the variances of angles in the free moving task were larger than in the following task because of the inter-subject variation in moving speed and range when there was no indicator. In addition, the Euler angle change in the static pattern was not shown here since the change was less than 0.5° in both moving tasks.

### Signal characteristics

**FIGURE 4:**
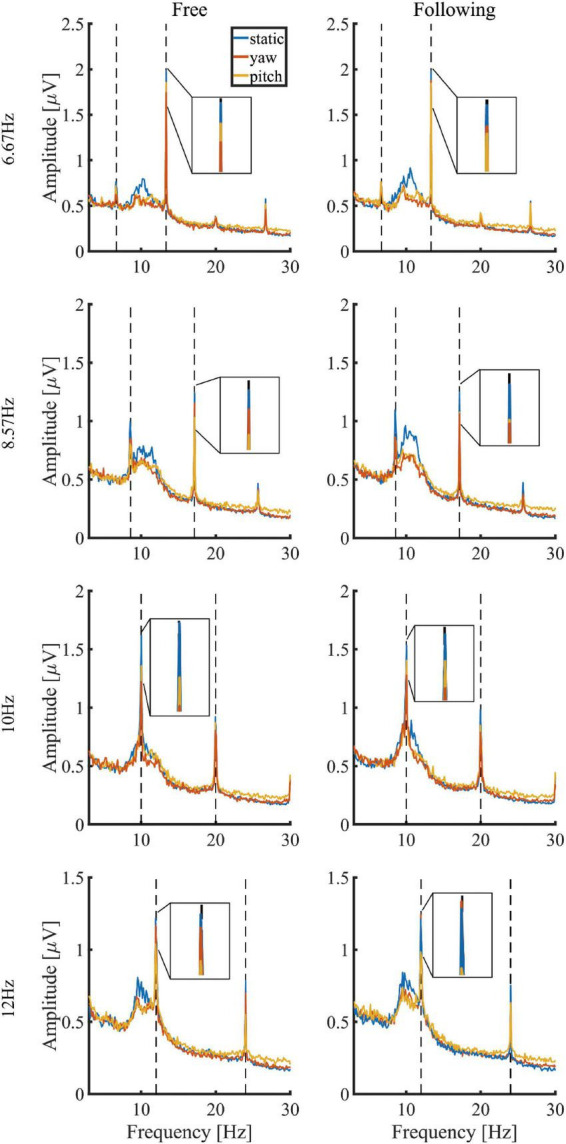
Comparisons of mean amplitude spectra in different moving tasks and patterns.

**FIGURE 5:**
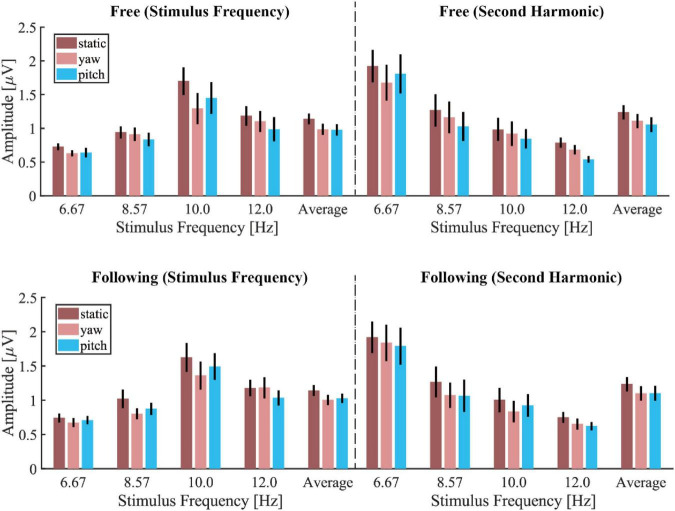
The mean and standard error of the mean (SEM) of spectrum amplitude corresponding to the stimulus frequency and its second harmonic in different moving tasks and patterns.

Figure 4 showed the mean amplitude spectra of different moving tasks and patterns under each stimulus frequency, and the mean spectra were averaged from all electrodes and subjects. The noise level in each condition showed no difference, but the stimulus responses differed. The SSVEP response in the static moving pattern displayed the highest frequency peak compared to the SSVEP in the other two patterns for both tasks. But there was no visible difference between the free and following tasks in each moving pattern. Figure 5 demonstrated the group average and standard error of the mean (SEM) of spectrum amplitude corresponding to each stimulus frequency and its second harmonic in different moving tasks and patterns. Under 6.67 Hz and 8.57 Hz visual stimulation, the frequency response of the second harmonic was higher than that of the stimulus frequency, but the trend that the static moving pattern elicits the highest SSVEP response didn’t change. The statistical results of four-way ANOVA showed significant difference in moving patterns [*F*(2, 30) = 6.776, *p* = 0.014, effect size = 0.311], frequencies [*F*(3, 45) = 7.289, *p* = 0.002, effect size = 0.327] but no significant differences in moving tasks [*F*(1, 15) = 1.260, *p* = 0.279, effect size = 0.077] and harmonics [*F*(1, 15) = 0.497, *p* = 0.492, effect size = 0.032]. The pairwise *post hoc* test of the moving pattern under all conditions showed that the SSVEP response amplitude of the static pattern was significantly higher than that of the yaw pattern (*p* = 0.037), but no difference was found between the static and pitch pattern (*p* = 0.059), yaw and pitch pattern (*p* = 1.000).

**FIGURE 6:**
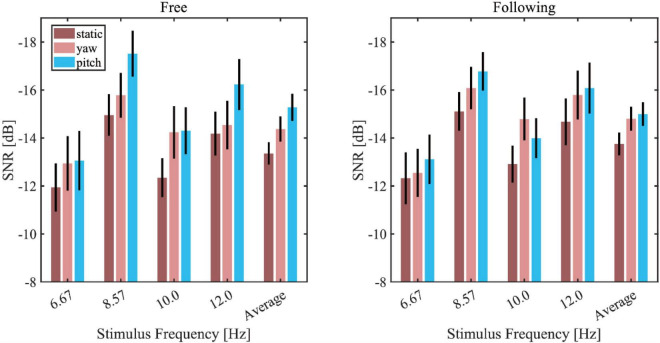
Wide-band signal-to-noise ratio (SNR) bar plot in different moving tasks, patterns, and stimuli (left column: free moving task; right column: the following moving task).

Figure 6 showed the average wide-band SNR bar plot with the SEM in different moving tasks, patterns, and stimuli. Because all the wide-band SNR was below zero, to visualize it intuitively, the vertical axis of the graph was reversed so that the higher the bar was, the lower SNR it represented. The static pattern had the highest SNR in all of the stimuli, and the yaw pattern in both moving tasks yielded the second-highest SNR, while the pitch pattern yielded the lowest SNR. In response to the 10 Hz stimuli, the SNR of the yaw pattern was lower than the pitch pattern, but generally, the average response showed a consistent trend with most frequencies. A three-way repeated-measures ANOVA was applied to test the effect of moving tasks, patterns, and stimuli frequencies. The results showed significance of difference in moving patterns [*F*(2, 30) = 9.360, *p* = 0.003, effect size = 0.384] and stimuli frequencies [*F*(3, 45) = 12.884, *p* < 0.001, effect size = 0.462] but showed no significance of difference in moving tasks [*F*(1, 15) = 1.049, *p* = 0.322, effect size = 0.065]. The pairwise *post hoc* test result of each moving pattern pair under all conditions revealed that the SNR of the static pattern is significantly higher than that of the yaw pattern (*p* = 0.041) and pitch pattern (*p* = 0.003). But there was no significant difference between the yaw and pitch pattern (*p* = 0.105).

### Classification result

**FIGURE 7:**
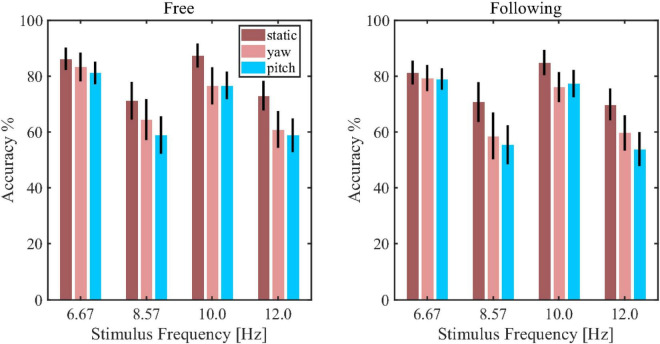
The decoding accuracy of steady-state visual evoked potential (SSVEP) using the canonical correlation analysis (CCA) approach in each stimulus frequency and moving pattern.

**FIGURE 8:**
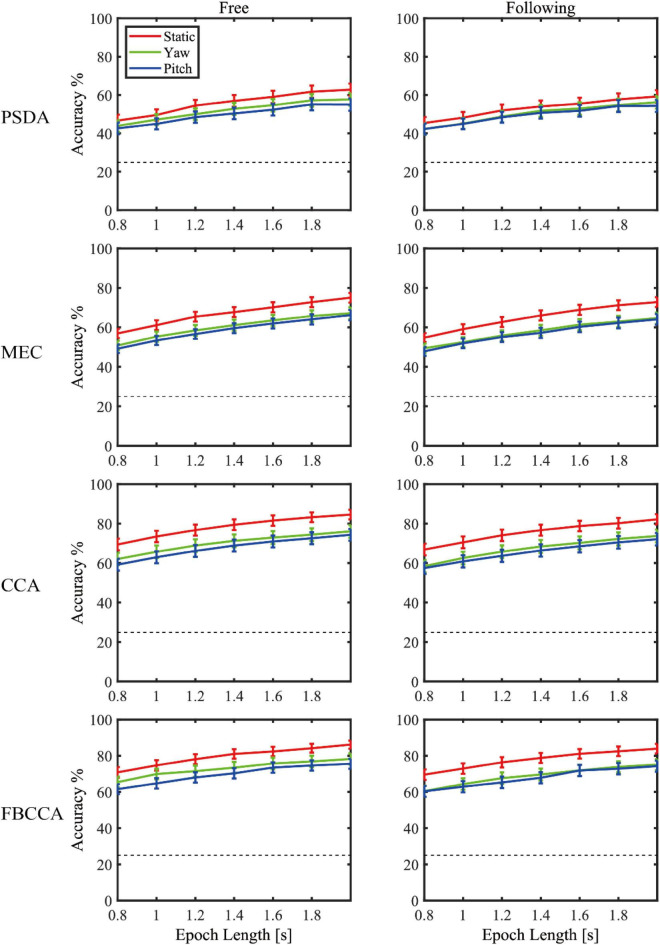
The decoding accuracies with SEM as varying the window lengths in both moving tasks for each algorithm.

Figure 7 showed the simulated detection accuracies with SEM as each algorithm varies the window lengths in both moving tasks. Table 1 listed the CCA classification accuracy of sixteen subjects under all conditions in 2 s epoch length. Furthermore, a four-way repeated-measures ANOVA was used by comparing the accuracies in a window length of 2 s. The test results showed significant difference in moving patterns [*F*(2, 30) = 9.296, *p* = 0.001, effect size = 0.383], frequencies [*F*(3, 45) = 6.327, *p* = 0.001, effect size = 0.297] and algorithms [*F*(3, 45) = 91.241, *p* < 0.001, effect size = 0.859]. No significant main effect was found in moving tasks [*F*(1, 15) = 3.314, *p* = 0.089, effect size = 0.181], which was in accordance with the test result of spectrum amplitude and SNR. The pairwise *post hoc* test of the moving pattern under all conditions showed that the accuracy of the static pattern was significantly higher than the other two [yaw (*p* = 0.047) and pitch (*p* = 0.003)]. However, the accuracy difference between the yaw and pitch pattern was not significant (*p* = 0.991). Besides, CCA and FBCCA algorithms obtained the highest accuracy compared to MEC (*p* < 0.001) and PSDA (*p* < 0.001). But there was no significant difference between CCA and FBCCA (*p* = 0.102). From the observations and statistical results, we could draw a few conclusions: (1) as the window length increased, the accuracies increased in all conditions and were distinctly higher than the 25% of chance level (the dashed line shown in Figure 8); (2) the classification accuracy of the static moving pattern was higher than the accuracies of the other two patterns in using all the time window lengths, which was in accordance with the previous results; (3) CCA and FBCCA algorithm demonstrated the best result, and PSDA showed the worst; (4) the classification accuracy in the free moving task showed no difference from the following task.

**TABLE 1:** The canonical correlation analysis classification accuracy of sixteen subjects under all the conditions in using the 2 s epoch length.

Subject	Task free			Task following		
	Static	Yaw	Pitch	Static	Yaw	Pitch
S1	**91.06**	74.98	72.34	**75.75**	71.66	70.86
S2	**84.81**	57.67	54.21	**79.80**	65.73	56.90
S3	**54.42**	37.72	48.81	**60.88**	36.42	42.13
S4	**82.00**	73.81	77.16	83.84	74.40	**84.96**
S5	**86.21**	83.41	74.43	**73.28**	68.43	60.13
S6	75.75	79.63	**81.36**	**73.28**	67.46	67.13
S7	**84.59**	39.53	48.28	**80.93**	35.87	50.94
S8	**97.52**	90.84	85.99	**97.52**	75.00	74.46
S9	**95.04**	90.35	85.54	90.84	**95.37**	88.36
S10	**99.89**	98.49	95.37	**98.60**	97.74	91.70
S11	97.20	98.10	**98.13**	94.94	94.40	**97.08**
S12	89.22	**89.69**	87.42	**88.79**	82.10	80.82
S13	**49.25**	33.08	42.93	**49.89**	49.25	40.63
S14	100.00	99.39	96.29	**99.46**	98.28	95.69
S15	70.83	**72.83**	54.30	65.30	**70.26**	60.92
S16	95.91	**97.00**	81.90	**99.46**	92.67	85.24
Mean	**84.61**	76.03	74.03	**82.04**	73.44	71.75
	**78.22**			75.74		

The bold values represent the highest value in each main class for each subject.

**FIGURE 9:**
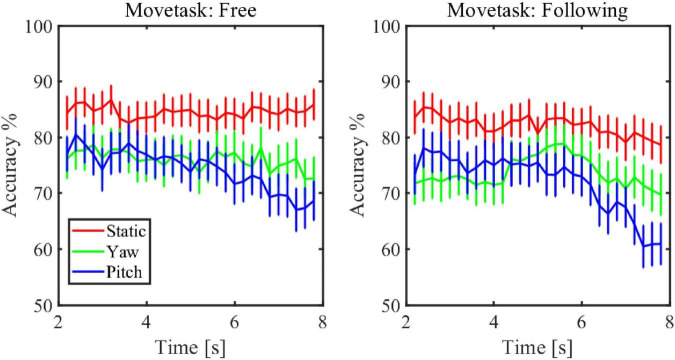
The change of classification accuracy across different time periods within one trial.

Furthermore, the changes of classification accuracy across different time periods within one trial were also explored in this study. The data was segmented using sliding windows and the CCA method was used to classify the result in each window. The window length was set to 2 s and the step time was 0.2 s. Figure 9 showed that the mean accuracy changed under all of the conditions. Because the result came out only after the data fills the window buffer out, the plot showed the change of accuracy from 2 s to 8 s. The figure showed that the accuracy of pitch pattern dropped most during the process of one trial. By applying the Mann-Kendall trend test, the accuracy of the pitch pattern had a significant downward trend in both moving tasks (*p* < 0.001).

## Discussion

In a realistic SSVEP-based BCI in the virtual reality environment, it is necessary for the operators to turn their heads and gaze at one of the stimuli simultaneously to interact with the surrounding environment better. It is hypothesized that head movement and the accompanying gaze fixation switch can decrease the performance of the SSVEP-based BCI. However, no studies were conducted to investigate this problem, and the effects of the factors on SSVEP responses and decoding accuracies were still unknown. This study proposed a novel ball tracking paradigm, and experiments were performed to compare both factors. In this experiment, three moving patterns were designed to simulate the actual head movements in real life. Moreover, two moving tasks were executed to compare the effects of accompanying gaze fixation change when moving the head. In the free task, the subjects directly gazed at the flickering stimulus all the time, while in the following task, the subjects might have to pay attention to the ball covertly while gazing at the stimulus. This gaze fixation point switch, could frequently happen in the realistic BCI. Thanks to VR technology, we could design an experimental paradigm to explore the head movements and gaze fixation switch simultaneously and separately. The current test results showed that only head movement could significantly decrease the performance of the SSVEP-based BCI, indicating that it was possible to gaze at the stimulus and observe the environment simultaneously.

In a previous study (Kanoga et al., 2019), the authors studied the effects of head movement in a non-VR scenario. The subjects tracked a single moving stimulus on a computer screen in their paradigm. The eye’s fixation was always in the center of the stimulus, making it unable to detect the surrounding environment. They reported that the amplitude and SNR of the SSVEPs decreased during the subject’s head movement, which were in accordance with our study’s results, as Figures 5, 6 showed. However, no significant differences were found between the yaw and pitch pattern after applying pairwise *post hoc* analysis in all of our results. The trend of SNR change agreed with the classification results, which meant that the higher SNR it was, the higher accuracy it yielded. Tracking a moving stimulus might be necessary for many practical BCI applications. The paradigm in this paper solved this problem by combing gazing at one of the four stimuli and tracking a ball together in a VR environment, which could give guidance in practical BCI applications like driving a wheelchair or controlling a robot arm.

Three popular training-free SSVEP classification methods, i.e., PSDA, MEC, and CCA, were adopted to compare decoding performance in different conditions. For each algorithm, the performance change due to head movements and gaze fixation switch showed consistency. The results showed that CCA and FBCCA outperformed the other two approaches in all experimental conditions and decoding window lengths. This was consistent with Lin’s (Lin et al., 2006) and Nan’s studies (Nan et al., 2011) in the static state. Moreover, Figure 8 and the statistical test also showed that CCA and FBCCA was better than PSDA and MEC under moving conditions, no matter whether the subjects tracked the ball or not. That was probably because CCA took advantage of the second harmonic. As shown in Figures 4, 5, the amplitude of the second harmonic was greater at 6.67 Hz and 8.57 Hz. However, the third harmonics were not higher enough and didn’t show better performance compared to the 2 harmonics in our pre-experiment. This might suggest that CCA was a more robust decoding algorithm for applications with inevitable head movement. Besides, the performance of FBCCA, one of the most popular SSVEP decoding algorithms, had been tested in this study. Although the mean numeric accuracy of FBCCA outperformed that of CCA, the pairwise *post hoc* analysis showed no significance between these two methods (*p* = 0.102). The reason could be that FBCCA was not more effective than CCA with a small number of stimuli and a large stimulus frequency interval. The first two harmonics could determine the final results mostly, thus *N*_*h*_ was selected 2 in the preliminary experiments. Besides, the high frequency noise over 30 Hz brought by head movement, could also affect the performance of FBCCA. Although FBCCA could be used as an unsupervised SSVEP decoding algorithm, in most cases, it still needs some calibration data for parameter optimization to get better performance in a specific system. Therefore, FBCCA could be took as an extension of CCA and might be useful in complex BCI with many stimuli.

During the process of one trial, the classification accuracy significantly dropped in pitch pattern. This could be caused by the complex force generation in pitch movement. Besides the visual fatigue caused by a long period of visual stimuli, subjects needed to activate their Sternocleidomastoid (SCM) more to overcome the influence of gravity during pitch movement, which meant that the SCM force to maintain the constant speed at different pitch angles was different. However, the head movement in the yaw pattern was perpendicular to the gravity, which meant that the SCM force to maintain the constant speed at different yaw angles was almost the same. As the experiment proceeded, subjects’ motion control became more and more difficult and could reduce their attention on the stimulus. Thus, the classification accuracy dropped.

Gaze fixation switch occurred when the gazed stimulus was not at the location of the tracked ball. Previous studies found a decreased trend when increasing the gaze distance from the stimulus center (Zhang et al., 2019). Gaze fixation switch happened when subjects gazed at the stimulus and tracked a target ball simultaneously; however, subjects could gaze at the stimulus directly in a free moving task. A primary objective of this study was to explore the effects of gaze fixation switch on the SSVEP response and classification accuracy; thus, the comparison between the two moving tasks was designed. The statistical significance test of all the amplitude spectrum, wide-band SNR, and offline accuracy showed no significant difference between the two moving tasks, which did not match our hypothesis. This might be due to a limited attention absence when subjects gazed at the stimulus most of the time and occasionally spared covert attention on the ball. The test results revealed that the gaze fixation switch caused by observing surroundings was not strong enough to deteriorate SSVEP-BCI performance significantly. Furthermore, a recent study (Meng et al., 2021) in our group also demonstrated a minimal influence of gaze fixation point for traditional motor imagery-based BCI. Therefore, it is feasible to design a realistic BCI application that requires more interaction with the environment than the conventional ones.

Notably, the background noise in the stimulus frequencies band showed no difference between the three moving conditions, as Figure 4 showed. This was not consistent with our proposed hypothesis. It might be because the spectrum of moving artifacts caused by the head movement was mainly distributed in frequencies higher than 30 Hz or lower than 3 Hz (Lin et al., 2013), which had been filtered out substantially in the preprocessing step. Besides artifacts, subjects have different mental workloads when performing these different moving patterns. Tracking a ball by moving the head required more cognitive effort and sensory engagement in processing the changing visual scene. A former study showed that the SSVEP accompanied by a simultaneous memory task could induce performance deterioration (Zhao et al., 2018). Lin et al. (2014) found that the alpha-rhythm could be inhibited by increasing visual processing during walking. The alpha-rhythm attenuation modulated by engaged cognitive and sensory tasks was known as alpha suppression (Williamson et al., 1997). In our experiment, the stimuli frequencies (6.67 Hz, 8.57 Hz, 10.0 Hz, and 12.0 Hz) were all located within the alpha-band. Because SSVEP could be regarded as phase and frequency locked EEG oscillations, it is reasonable to suppose that the decreased SSVEP was caused by reduced alpha rhythm. The amplitude spectra in Figure 4 could also explain that the alpha-band amplitude in the static moving pattern is distinctly higher than in the other two patterns. Therefore, the decreased SSVEP performance with the different head movement patterns might not be caused by additional artifacts but by additional mental workload and induced alpha suppression. However, whether training could reduce this detrimental effect on SSVEP might be worth further investigation (Wan et al., 2016; Meng and He, 2019).

Although this study tried to design an experimental paradigm that was as close to the actual application as possible, there were still some limitations. First, the head movement was simplified to two moving directions and one set of several representative speeds. The roll movement was eliminated because this moving pattern occurred less commonly when a person tried to expand the view and explore the surrounding environment. The speed was compromised to one gear to control the number of trials. Therefore, there were still some gaps between the experimental design and the actual application scenarios. But since the complicated movements could be considered a combination of yaw and pitch movements, this study was still instructive for the practical movements to a certain extent. Second, gaze fixation switching happened in this experiment which caused variation in visual attention, but there was no objective measurement of selective attention in this study. Thus, selective attention was not quantitatively controlled. In this sense, it was possible that even when the stimulus is not moving and people gaze at a stimulus, subjects did not necessarily pay overt attention to the stimulus although they were instructed to do so. Therefore, some kinds of objective measurements are needed to investigate the effect of visual selective attention. For example, in Xu et al. (2016) subjects were asked to press a button when they realized the changes in stimulus where they were paying covert attention. In future research, we would like to test the influence of visual selective attention in a rigorous way. Third, this experiment mainly focused on offline data analysis, and the online experiment was not performed. Therefore, it is worth further assessing the online decoding performance of the SSVEP-based BCI applications with inevitable head movements such as wheelchair control.

## Conclusion

This study proposed a novel ball tracking paradigm to investigate the influence of head movement and gaze fixation switch on SSVEP responses and classification accuracy. Sixteen subjects were recruited for this experiment. The offline data analysis indicated that only the head movement could decrease the performance of the SSVEP-based BCI while the changed visual gaze fixation did not. Furthermore, the CCA and FBCCA decoding method showed more robustness than PSDA and MEC. This study suggested that head movement was one of the critical influencing factors on the SSVEP-based BCI, and further improvement has to be made in future applications.

## Data availability statement

The raw data supporting the conclusions of this article will be made available by the authors, without undue reservation.

## Ethics statement

The studies involving human participants were reviewed and approved by the Institutional Review Board of Shanghai Jiao Tong University. The patients/participants provided their written informed consent to participate in this study.

## Author contributions

JD and JM wrote the first draft of the manuscript and conceived and designed the experimental paradigm. JD, SL, NZ, and JM performed the research and analyzed the data. JD, LL, NZ, and JM wrote the manuscript. All authors edited the manuscript.
